# Modifications in the Topological Structure of EEG Functional Connectivity Networks during Listening Tonal and Atonal Concert Music in Musicians and Non-Musicians

**DOI:** 10.3390/brainsci11020159

**Published:** 2021-01-26

**Authors:** Almudena González, Manuel Santapau, Antoni Gamundí, Ernesto Pereda, Julián J. González

**Affiliations:** 1 Departamento de Historia del Arte (Música), Universidad de La Laguna, 38200 Tenerife, Spain; 2 Conservatorio de Música de Requena, 46340 Valencia, Spain; santapaucello@gmail.com; 3 Departamento de Biología, Universidad de las Islas Baleares, 07122 Palma de Mallorca, Spain; antoni.gamundi@uib.es; 4 Grupo de Ingeniería Eléctrica y Bioingeniería, Departamento de Ingeniería Industrial e Instituto, Universitario de Neurociencia, Universidad de La Laguna, 38200 Tenerife, Spain; eperdepa@ull.edu.es; 5 Departamento de Ciencias Médicas Básicas, Fisiología (Medicina), Universidad de La Laguna, 38200 Tenerife, Spain

**Keywords:** graph-based analysis, complex networks statistic, music styles

## Abstract

The present work aims to demonstrate the hypothesis that atonal music modifies the topological structure of electroencephalographic (EEG) connectivity networks in relation to tonal music. To this, EEG monopolar records were taken in musicians and non-musicians while listening to tonal, atonal, and pink noise sound excerpts. EEG functional connectivities (FC) among channels assessed by a phase synchronization index previously thresholded using surrogate data test were computed. Sound effects, on the topological structure of graph-based networks assembled with the EEG-FCs at different frequency-bands, were analyzed throughout graph metric and network-based statistic (NBS). Local and global efficiency normalized (vs. random-network) measurements (NLE|NGE) assessing network information exchanges were able to discriminate both music styles irrespective of groups and frequency-bands. During tonal audition, NLE and NGE values in the beta-band network get close to that of a small-world network, while during atonal and even more during noise its structure moved away from small-world. These effects were attributed to the different timbre characteristics (sounds spectral centroid and entropy) and different musical structure. Results from networks topographic maps for strength and NLE of the nodes, and for FC subnets obtained from the NBS, allowed discriminating the musical styles and verifying the different strength, NLE, and FC of musicians compared to non-musicians.

## 1. Introduction

Contemporary academic music emerged in Europe in the early twentieth century in a break with the structural bases of previous styles of music built from the Renaissance such as tonality (harmony) and rhythm, and incorporating new sounds, effects, musical syntax, definitely a new stylistic idea. The present work addresses an investigation about how the flow of neural information between brain networks is comparatively reflected during the listening of one contemporary atonal music excerpt of stylistic new features and ruptured structure, a listening of one baroque music excerpt as a paradigm of melody governed by metrical/rhythmic and tonal-harmonic structure, and a listening of pink noise, sound containing all audible frequencies/pitches with constant intensity/sonority. About the latter, it is known that the cortex electrical activity appears to be sensitive to alterations on pitch frequency and the perceived metric [1,2]. The study will be carried out by using properly electroencephalographic (EEG) techniques, the analysis of the transfer of neural information among cortex networks.

The cognitive process of musical perception allows us to interpret the musical sensation and respond with different types of emotions and expectations. In this sense, it has been related that interaction with music is a continuum between the sensory and the cognitive [3]. However, musical emotionality will depend on musical syntax and we know that the differences between musical structured styles concern musical syntaxes, established by the tonal system through harmony and defined rhythmic patterns. It has been related that musical tension syntax base is generated through sequential and hierarchical harmonic use in musical passages, which produce the psychological responses associated with them [4]. This concept of musical tension is related to the processing of the intra-musical structure by means of underlying structural factors [5]. In this regard, several studies [2,6,7] have indicated that the intervention of defined rhythmic patterns accompanying music tonal structure can trigger cortical synchronization or entrainment phenomena, leading to a continuous generation of expectations and predictions [8,9] and/or sense of anticipation [10]. These psychological responses appear to be closely linked to the human perception of tonal music [11]. It has been pointed out that some contemporary styles break with the ordered structure and it has been addressed from the field of experimental psychology. Thus, it has been postulated that dissonant inharmonic music interferes (some styles of contemporary academic music use this feature) with cognitive performance, requiring greater cognitive processing than harmonic music because of its greater acoustic complexity and irregular syntactic structure [12]. These musical characteristics have been reported to produce a greater sensorial complexity of unexpected and disconcerting situations, or moments of unfulfilled expectations and higher levels of arousal [13]. The alteration in cognitive performance by dissonant intervals is, however, under discussion by other authors [14]. In addition to factors linked to musical syntax, musical perception is modulated by factors associated with the listener’s personal characteristics: age, cultural level, socio-economic and cultural context, musical experience and learning, familiarity with the type of music heard, psychological state, and preferences [15,16,17]; in short, all the factors that define the degree of enculturation [18].

Functional magnetic resonance imaging (fMRI) studies show that different emotions and sensations caused by music are controlled by different areas of the brain located in the limbic and para-limbic system associated with control/modulation of emotions and reward mechanisms [5,19,20]. Moreover, from the perceptual/cognitive aspect, listening to music is known to induce various actions: motor, social, of recognition, reflection, identity, or predictability, which involve trunk-encephalic areas related to the motor system, memory, multimodal integration, attention, or learning [5,21]. But EEG techniques have been considered very useful for analyzing cognitive and emotional musical perception over relatively long periods of time [22,23,24]. Thus, EEG spectral power measurements from different cortical areas appear to indicate that musical processing may entail local and/or distant neural networks whose communication may affect the power of different EEG frequency bands [25,26,27,28]. In addition, depending on the music style heard, different alterations in EEG spectral power occur in different bands and only in certain cortical areas [29,30,31]. Moreover, EEG measures of interdependence between different cortical regions have also been used in brain research, since cognitive activity is generally known to require different brain regions to co-act simultaneously, and a functional interaction takes place between them [32]. In this respect, frequency EEG oscillations have also been considered crucial for linking different elements and merging them into a coherent percept relevant to the processing of music considered as a multifunctional stimulus [33]. Recently, different measures of EEG functional connectivity (FC) have led to the association of some musical emotions with different patterns of neural correlations in some EEG frequency bands on the pre-frontal cortex [34], the correlation of different types of music with different emotions [35], or the differentiation between unknown music and familiar music [36].

One important factor through which music audition and brain processing can be better understood is musical expertise in order to clarify differences between the perception of musical styles, enculturation phenomena. Thus, the use of magnetic EEG oscillations showed that a measurement of signal phase information of gamma oscillations was enhanced in music experts versus non-musicians when they listened to dissonant chords [37]. Additionally, theta and gamma EEG activity of posterior cortical regions decreased with musical experience during the audition of major and minor compositions [38]. Using FC procedures, audition of tonal music excerpts modified EEG spectral coherence magnitudes in alpha and beta bands in musicians versus non-musicians [39]. Moreover, also what happened in the gamma band phase synchronization and in musicians was different than in non-musicians listening to the same musical excerpt [23]; and through EEG FC phase coherence measures, it was reported that FC increases in the alpha band during Chinese music audition in expert musicians [40]. Therefore, music expertise clearly influences single and synchronization EEG measures.

Other procedures use FC measurements between two regions/signals (fMRI or EEG) to obtain the connectivity matrices (connectome) between the set of brain regions of interest (case of the fMRI) or between the set of recording electrodes/regions of cortical activity (EEG case) and derive from them, by means of appropriate mathematical procedures, the neural networks involved in a certain condition. In addition, considering the graph formed by the values of the FC matrices as edges and the brain regions considered as nodes, different measures of graph theory are used to obtain parameters about the topological behavior of the networks. Recent studies of EEG or magnetoencephalography ( MEG) FC that make use of graph metrics to assess the levels of topological organization of brain networks have been shown to be useful in the analysis of dynamics and cognitive functioning [41,42], assessment of neurological dysfunctions such as dyslexia [43], neurodegenerative dementias and Alzheimer’s [44], schizophrenia [45], or depression [46]. In the brain-musical research context, it has been reported that music audition affects the topological structure of brain networks producing a trend for random network organization, thereby indicating a move to a more efficient but less economical architecture during music audition [40].

Our hypothesis is that the listening to breakthrough styles of atonal contemporary music produces different responses than those produced during listening to previous academic styles in the neural information transfer between certain cortical networks and conditioned by the cognitive musical experience. We will use EEG FC measures between different cortical areas based in the phase synchronization, among them computed in non-musician and musicians’ subjects. With these FC measurements obtained during listening tonal and atonal excerpts and pink noise, the techniques used to demonstrate the hypothesis were: (a) network-based statistic to identify connections and networks comprising the connectome and, (b) graph theory metric to analyze information transfer among networks. Additionally, the results will be discussed according to the musical structure of the sound stimuli and according to certain psychoacoustical spectral parameters related to the tone-color (timbre) characteristics of such stimuli.

## 2. Materials and Methods

### 2.1. Groups and Sound Stimulus

#### 2.1.1. Participants

A total of 32 volunteers, aged between 32 and 58 years, were available. They were divided into two groups according to their level of musical expertise: 16 professional musicians (M) of different musical instruments, mean age (±SD) 44.25 ± 8.95 (eight males and eight females), and 16 with no musical experience (NM), mean age 44.07 ± 9.31 (nine females and seven males). The level of musical expertise was determined by music training intensities in time and by professional career. An expert musician should have more than 20 years training and experience in musical practice and active professional practice. The selected NMs were only occasional listeners of classical tonal music (less than 10 auditions per year) and although they were aware of the existence of atonal contemporary music, they declared they did not like it and therefore did not listen to it.

All participants were right-handed (confirmed by the Edinburgh questionnaire), with no history of neurological disorders, and normal hearing. Participants underwent a previous EEG to rule out any electrographic signs (k-complexes, epileptic discharges, etc.) of neurological dysfunction. Participants received verbal and written information about the type of study and were previously provided with a document to sign their consent. The Ethics Commission of the University of La Laguna, Tenerife, Spain approved the protocol with the Ethic Approval Code CEIBA2014-0098 in accordance with the ethical standards of the Declaration of Helsinki, and all volunteers consented to participate.

#### 2.1.2. Sound Stimuli

There were three types of sound stimuli or auditions: the first (T) consisted in the 26 s of a tonal excerpt (T) of Sarabande (II Suite for solo cello, J.S. Bach); the second was Synchro, a transformation of Sarabande composed by one of the authors (A. González) with contemporary atonal (A) music characteristics (same duration as T); and the third, 26 s of pink noise (N) (files sounds are in Supplementary Materials). T and A are naturalistic stimuli, recorded by a professional cellist (see audio in complementary attachments). Stimuli (T and A) were selected because they belong to two different musical styles. T is a baroque excerpt composed in a tonal structure, and with a defined rhythm and metric parameters. During the 26 s of T, the musical line goes through several degrees within tonality (e minor), returning to tonic at the end with defined metric parameters. It was chosen because it meets the requirements of expectation, anticipation, and predictability of the tonal structure and defined rhythmic patterns [8,10,47]. The transformation of the first stimulus (T) into the second (A) stems from the same note, though A has no tonality, in order to maintain tessitura and gradually add effects and elements characteristic of the new music, including free parameters of metric and tempo, atonality, microtones, pizzicatos left hand, glissandi, and overpressure. The composition process of A was as follows: the two excerpts T and A are constructed in parallelism, as follows: I (a) Degree (tonic) {T and A excerpts begin with the base note of the tonality (D minor)}; V (a) Degree (dominant) {the second most important tonality degree is reached in both excerpt, and in T, its function is to give emphasis and tension to the music (common in baroque music); in A, it keeps this degree but makes ascending and descending glissandi, simulating the effect of T trill but in a contemporary language}; I (b) (tonic) Degree {in T, the tonic comes again to give distension to the music; in A, in order to keep a similar tessitura to T}; Bridge (a) {transition between different musical phrases in T, using the joint notes between the F of the first degree and the Bb of VI degree, and in A, more adorned and extended by quintuplets, the semibreve D and the crotchet C}; Degree VI (superdominant) {T excerpt reaches this degree, whose function is to generate a slight harmonic tension, in A to maintain tessitura and similarity}; Degree IV (subdominant) {its function is to move toward the dominant (V (b) Degree), T uses passing tone and A with the notes Bb and D that form, together with G, even if it is not present, the subdominant chord}; Degree V (b) (dominant) [as above, the previous subdominant is directed toward the dominant, represented by the third of the fifth degree (C#) and reinforced by the trill; A uses a micro trill over the C note, creating a similar effect]; bridge (b) {both T and A use the same compositional technique, forming a set of descending joint notes that flow, in the case of T to the I Degree, while in A it ends in a strange note outside the main tone and with overpressure effect]. The subjects do not know that A derives from T, and they are not familiar with the piece because they have never heard A before. The third stimulus N consisted of hearing 26 s of pink noise, which is taken as a control sound because it has an approximately constant spectral power in the octave interval filled by the stimulus spectra A and T.

#### 2.1.3. Acoustic Properties of Sound Stimuli

The three sound signals were analyzed in order to represent their temporal evolution over the 26 s and to quantitatively contrast some indices related to their acoustic behavior. The temporal evolution of the power spectra of stimuli was assessed throughout the time-frequency spectra representation of each one. This was carried out by computing the MATLAB© spectrogram function for which signals-stimuli were sampled at 44,100 Hz. A spectrogram was obtained for successive hamming tape windows of 8192 samples, overlapped in 60% of samples, until 26 s were complete. The 26-s period was then divided into 13 segments of 2 s each, and for the ensemble of spectra of each segment, the following indices were calculated:

(A) The mean and the variability, assessed by the standard deviation (SD) of spectral centroid (SC) between frequencies 60 and 4000 Hz, corresponding to octaves 2 to 7 of the piano, the interval in which most of the harmonics produced by notes or chords played by a cello are found. The SC indicates the mean frequency or center of mass of the spectral frequency interval analyzed, is considered a good predictor of the timbre “brightness” of a musical sound, and has been used for assessing the perceptual evolution of musical timbre [48,49]. In our context, SC variability allows us to know the evolution of timbre brightness throughout the 26-s sound auditions.

(B) The mean and SD of the spectral entropy (SE) of stimulus spectra in each successive two-second segment. The SE is calculated from the power values of the normalized spectral coefficients and by applying the Shannon entropy formula of the information theory. Spectral and wavelet entropy measurements [50] have been used to estimate the complexity and predictability of nonlinear signals. Thus, we use the SE as a measure of the timbre complexity/predictability of sound stimulus and it allows us to study its evolutions over the 26 s. In a piece of tonal music, expectation about what will probably come next often increases as the piece progresses. Normally at the end of the work—change from the dominant to the tonic—is often very predictable because of its conclusive nature. In entropic terms, final phrases entropy is lower than that of beginning phrases [51]. The evolution of SE can therefore provide us a quantitative measure of the entropy/predictability during the ongoing stimuli T and A. Finally, from the 13 means and 13 standard deviations of SC and SE, the global average of the means and the global average of the standard deviations were computed. SC and SE mean and variability will subsequently be used to assess alterations with sound stimuli in the topological indexes of EEG functional networks of subjects.

### 2.2. Acquisition of EEG Signals

EEG signals were acquired using a Nihon Kohden P-electroencephalograph and 19-channel monopolar EEG recordings were made for each subject (Fp1-2/F3-4/F7-8/C3-4/T3-4/P3-4/T5-6/O1-2) firstly referenced to the average of the reference electrodes A1 and A2 placed at the mastoids following the standard EEG system 10–20, and secondly re-referenced with the reference at infinity and the potential at zero or a constant, using reference electrode standardization techniques (REST) [52,53] and the scripts of REST Toolbox (http://www.neuro.uestc.edu.cn/rest/). EEG signals were filtered online with a low and high pass band, with frequency cutoff at 80 and 0.05 Hz, respectively, and a notch filter at 50 Hz. Signals were sampled at 500 Hz and the impedance of the electrodes was controlled to remain within 3–5 k-Ohm range. In addition to EEG, electrocardiogram (ECG) and abdominal breathing movements were recorded for the subsequent detection of artifacts and episode visual selection. All digitized records were stored in the computer for further preprocessing and analysis. The records were made at rest, with the subject comfortably seated in a chair, eyes closed, and wearing a sleeping mask, in an acoustically and electrically isolated room (Faraday cage), with the lights off. The sound stimuli (monophonic) were presented to the subjects through two loudspeakers located 2 m in front of their head, 50 cm apart, outside the Faraday cage. The decibel level of the T and A stimuli oscillated in a range of 50–80 dB and the N close to 60 dB. We used COGENT software in MATLAB language to successively present the 26-s stimuli (each stimulus was repeated ten times at random) together with the EEG record. Before the experiment began, participants were told that any movement or muscular tension would compromise the EEG signal and, for that reason, they were asked to avoid blinking, movement, or muscle tension.

### 2.3. Analysis

1. Preprocessing

(a) In order to avoid transition effects between stimuli, the EEG data of the first four and the final two seconds of stimuli were disregarded for the analysis. Therefore, for each subject, 40 EEG episodes of 5 s per stimulus were available. The EEG episodes corresponding to the same subject and auditory stimulus (T, A, or N) were first selected by visual inspection, in order to disregard those clearly contaminated by artifacts, using the ECG and the simultaneous respiratory signals. (b) The remaining episodes were detrended and subsequently normalized to zero mean and unit variance. (c) They were then arranged according to their stationarity level, using the Kwiatkowski–Phillips–Schmidt–Shin (KPSS) test for stationarity [54], as described in a previous work [55]. For this procedure, the twenty most stationary of all those available for each stimulus and subject was selected. With this procedure, we intend to select the artifact-free, more homogeneous and more similar segments that are indeed representative of the EEG activity generated in the experimental study condition. (d) Finally, and prior to the estimation of synchronization between EEG channels, the selected segments of each EEG episode were filtered using a finite impulsive response (FIR) filter of zero phase distortion (filter order: 256) in the following five frequency bands (FBs): delta-δ (0.1–4 Hz), theta-θ (4–8 Hz), alpha-α (8–13 Hz), beta-β (13–30 Hz), and gamma-γ (30–48 Hz).

2. Measuring EEG functional connectivity y (FC)

For assessing the FC between EEG channel pairs, we used the so-called phase-locking value (PLV), a phase-synchronization (PS) index, which has been shown to be sensitive to changes in the activity of deep sources and to the interdependence between them [55]. Its usefulness has also been contrasted against other PS indices to assess possible changes in the topological organization of brain networks, using graph metrics in studying disconnectivity in neurological disorders [45]. The steps for computing the PLV between two noisy x_k_(t), x_j_(t) real-valued signals are briefly as follows: First, the phases of each signal are estimated by constructing the analytic signals of the two narrow band signals, using the Hilbert transform. From this, the phase signals of x_k_(t) and x_j_(t) are obtained and the relative phase between them (φ_kl_ (t)) calculated. Finally, the PLV is calculated as the modulus of the complex exponential constructed using φ_kl_ (for a precise mathematical procedure, see [45,55]. MATLAB script for PLV computation can be found in HERMES toolbox (http://hermes.ctb.upm.es/). Two-second windows with 50% overlapping for each 5-s stimulus trial were used to obtain PLV. Extreme values for PLV are 0, indicating PS absence, or φ_kl_ uniformly distributed, or 1, when total PS exists or φ_kl_(t) is constant.

PLV can be affected by common noise brain sources (like volume conduction) or by characteristics of signals other than the existence of statistical relationships between them. Therefore, it would be convenient to have a test that assesses the statistical significance of the interdependence (PS) provided by the PLV as an index of the FC. In addition, as we will comment later, the indices of graph theory are usually calculated using only a percentage of the set of inter-electrode connectivities (those that exceed a predetermined connectivity threshold). Here, a surrogate data test is used to verify the real interdependence of PLV. First, 100 surrogate versions, which preserve all their individual features (amplitude distribution, power spectrum) but are independent by construction, are obtained for each channel signal. Surrogate signals were generated using the twin surrogate algorithm [56,57,58], which has proven useful when some signals are affected by nonlinear traits, a question that cannot be ruled out in the case of EEG data [59,60]. The twin algorithm used the signal’s recurrence plot, requiring for operation the appropriate reconstruction of the state space of the systems that generate the signals. To perform this operation, appropriate embedding dimension and time delay are needed. Here, they were estimated by the false nearest neighbor procedure [61] and mutual information function, respectively. For each, the PLV index between two channel-signals, named k and j, the PLV_k,j_, is obtained; then, 99 PLV indices between the original xk and each of the 99 x_js_ surrogates (PLV_k,js_ (s = 1, …. 99) are computed. From this, the empirical distribution of PLV values is obtained under the null hypothesis of PS absence. Finally, the original value of the index (PLV_k,j_) was considered significant, at the *p* < 0.01 level, if PLV_k,j_ > PLV_k,js_ for any s; when this does not occur, PLV_k,j_ is set to zero.

3. Graph-based analysis

To adapt the theory of graphs to a cerebral neural network, taking into account the cortical measurements of electroencephalographic FC at each FB, the nodes are the EEG channels placed at 19 cortical brain regions, and the 18 inter-channel undirected FCs per node are the links or edges between nodes. In this way, 19 × 19 weighted adjacency undirected matrices (AW) of FCs are arranged for graph metrics. In fact, for each FC threshold, there are five AW matrices per condition (stimulus), subject, and FB for subsequent computing of graph metrics.

4. Topological measures of EEG-FC networks

From each AW matrix, the following central topological indices of each EEG-FC network were obtained for each node and then averaging for all nodes: (a) node density equal to the fraction of present (non-zero) FCs to possible FCs and (b) node strength taken as the mean of non-zero FCs linked to each node. With regard to the topological inter-node organization of the EEG network, the following graph metrics were computed from each Aw: the global efficiency (GE); it is a measure of the ability of the graph to interconnect and transmit information between distant nodes. For a graph (G), GE is defined as the average of the inverse of the shortest path length from each node to all other nodes [62,63] which is mathematically expressed by:

$$\mathrm{GE}\left(G\right)=\;\frac{1}{N\left(N-1\right)}\;\sum_{i\neq j}\frac{1}{d\left(i,j\right)},$$

where d(i,j) is the shortest path length between node i and node j in graph G and is calculated as the smallest sum of edge/connections lengths throughout all possible paths from node i and node j. The length of a connection or edge was considered as the reciprocal of the connection weight (here, their PLV or FC), under the assumption that the distance between two nodes is inversely proportional to their FC [64]. Local efficiency (LE), which for a node is defined as the GE of the node, calculated in the subgraph created by its neighbors. The LE of the graph (G) is the average of the LEs of all nodes. It is calculated by applying the same steps in the subgraph (Gi) formed by the neighbors of node i:

$$\mathrm{LE}\left(G\right)=\;\frac{1}{N}\;\sum_{i∋G}\mathrm{GE}\left(G_{i}\right).$$

Taking both measures (LE and GE) into consideration, a regular network or lattice is defined as having high LE and low GE, a random network has low LE and high GE, and a small-world (SW) network would lie somewhere between a regular and random network, with a high LE and GE. To check whether a real network has a small-world structure, one of the proposed methods is to normalize LE and GE in the real network, in relation to those computed in matched random networks e.g., dividing LE and GE by the corresponding mean [LE(r) or GE(r)] obtained from 100 random networks that preserved the same number of nodes, connections, and degree distributions as the real brain networks [64,65,66]. In this way, a SW network would be characterized by having LE > LE(r), and a comparable GE ~ GE(r) or having the normalized versions NLE = LE/LE(r) > 1 and NGE = GE/GE(r) ~ 1.

The young adult brain appears to have a SW-like architecture, its functional connectivity consisting of groups of brain regions with strong local connections to each other, along with some global connections between these groups in the form of neural centers. On the other hand, with aging, there do not seem to be substantive changes in *LE* with respect to young adults, but there do appear to be lesser *GE* in relation to the number and organization of functional connections, which imposes higher costs for the transfer of information with the aging [67,68]. MATLAB Brain Connectivity Toolbox (http://www.brain-connectivity-toolbox.net) [69] was utilized to calculate the previous indices from the graph theory.

The SW structural parameters of brain graphs such as LE/GE is known to be influenced by the magnitude of the graph degree or density. Consequently, when comparing different groups/conditions, differences that may be found in the SW topological indices could be attributed—or biased—by the differences in the degree/density. For this reason, it has been suggested that topological indices be computed at different node-degree or density thresholds and each analyzed for possible differences in the topology of the network [70]. In this study, the LE and GE indices of a network have been calculated using the matrix of FCs resulting from applying the surrogate data test to the synchronization indices between nodes (PLV) that corrects or threshold, as we have seen, the graph degree as a function of the statistical significance of those indices. In accordance with the above, it is always necessary to take into account the changes in the degree to density of a graph when comparing different groups or situations basing on alterations in their topological graph properties LE or GE.

5. Statistical analysis of stimuli acoustic features

The homogeneity of the variances of the means of SC and SE in the 13-s segments of the 26-s stimulus [M-M-SC, M-M-SE, M-SD-SC, and M-SD-SE] was verified with the Brown–Forsythe (BF) test. In the absence of homogeneity, the Kruskal–Wallis nonparametric test (K-W) was applied to compare the means of the three stimuli or the ANOVA test. Then, for post-hoc comparisons between pairs of conditions, we used the Mann–Whitney U test (MWU), or *t*-test with Bonferroni correction.

6. Network-based statistic (NBS)

Starting from the connectivity matrix of a graph, this technique aims to determine statistically if between two groups/conditions between which a contrast hypothesis is established, a subnet of FCs is formed between different brain regions that confirms/rejects the existence of the hypothesized difference. This analysis was carried out through the MATLAB toolbox (NBS toolbox, https://www.nitrc.org/projects/nbs/) [71] that implements the so-called statistics network-based (NBS) and other statistical methods to test hypotheses about the human connectome. NBS is a non-parametric statistical method based on permutation of the compared groups/conditions (e.g., by *t*-test) and uses the FWER correction (family wise error rate) to solve the problem of multiple comparisons in a graph and to be able to select subnets formed by connections whose weights are significantly different between groups regardless of whether their weights/connectivities are strong or weak [71].

To operate with the NBS toolbox, first of all, we prepare/order the connectivity matrices of each subject in each group or condition: in this work, connectivity matrices of 19 × 19 elements. Next, the two groups of matrices to be compared are concatenated, the appropriate contrast vector is prepared and in the case of paired comparisons, the pairing matrix (DesigMatrix) and the exchange matrix are designed for the selection of the order of the permutations (ExchgBlock). We then select the appropriate *t*-test (paired or unpaired) of contrast between the groups/conditions and a threshold value for the t statistic (see the NBS reference manual) to limit the set of supra-threshold links used by the NBS. In addition, we must choose a suitable number of permutations M (normally between 1000 and 5000) to estimate the null distribution of the maximum size of the component (grafo’s subset of connections) and the magnitude associated with the component of the graph to be measured, that is, its extension (number of connections) or either its intensity (sum of T values). Finally, we must choose a limit value of *p* (normally a value of 0.05 or 0.01 for the FWER) to limit the distribution of *p* values obtained from the M permutations/comparisons. The NBS will provide us with the mean *p* of the distribution that is less than the selected limit; this allows us to confirm/reject the existence of a subnet with higher/lower connectivity links for the control group/condition in relation to the other experimental group/condition.

7. Permutation test for comparisons between the nodal indices of two graphs

We use the MATLAB routine “mult_comp_perm_t1” and “mult_comp_perm_t2 (https://www.mathworks.com/matlabcentral/fileexchange/29782-mult_comp_perm_t1-data-n_perm-tail-alpha_level-mu-reports-seed_state; https://www.mathworks.com/matlabcentral/fileexchange/29782-mult_comp_perm_t2-data-n_perm-tail-alpha_level-mu-reports-seed_state) [72,73] to compare at the nodal level the difference between the matrices—which columns are the values of a certain index of the graph (degree, strength, or NLE) in the 19 nodes of the graph and which rows are the index values for each of the group member (*n* = 16)—computed during two different auditions or groups. This procedure is equivalent to comparing multiple repeated variables and concerns the statistical issue of multiple comparisons with the problems of false positives that occur in this type of comparisons. As in the NBS, a permutation test of the null hypothesis is used that a set of data was sampled from a symmetric distribution with a particular mean. The test is based on the repeated measures *t*-test and is more general than parametric *t*-tests, as it does not assume that the data were sampled from a Gaussian distribution. This method adjusts the *p*-values in a way that controls for the family wise error rate (FWER). However, the permutation method is more powerful than the Bonferroni correction when different variables in the test are correlated. For the operation of the test, see the toolbox [72] or [73]: we use 10,000 permutations for an alpha level of 0.001. As output data, the MATLAB routine provides an adjusted *p*-value for multiple comparisons of each repeated variable (pval), and also the estimated alpha level for the FWER value (est_alpha).

8. Statistical analysis of graph/network indices

We used MANOVA for repeated measurements to check the existence—in each of the FB EEG connectivity network—for the global graph indices of (a) between-groups differences (MNM) regardless other factors, (b) differences between the three auditions (TAN) or five FBs regardless other factors, (c) conditions interactions (FB*TAN) regardless of groups, and (d) group-conditions interactions (TAN*MNM, FB*MNM). MANOVA test gives in contrast (a) the contrast the (F) statistic and level of significance (P). The MANOVA statistic steps (b), (c), and (d) were performed by a multivariate test including Wilks’ and Roy’s test (W-R). Mauchly’s test of sphericity was considered when the within-effects table showed significant differences for any of these (W-R) (*p* < 0.05 or lower). If the condition of sphericity did not hold, we used the adjusted univariate tests for repeated measure, using the lower-bound (L-B) test for the F statistic estimation. According to the number of groups (2) and subjects (16 NM, 16 M), the appropriate degree of freedom (DF) is taken into account in each case. Finally, when an effect was statistically significant (*p* < 0.05, *p* < 0.01 or *p* < 0.001), the post-hoc Bonferroni test was applied for pairwise comparisons between repeated factors.

Several MATLAB function for topographic plots of inter-nodes connectivities as “f_PlotEEG_BrainNetwork” (https://www.mathworks.com/matlabcentral/fileexchange/57372-easy-plot-eeg-brain-network-matlab) [74] and the Matlab “topoplot” for plotting node values of graph indices, or the level of their statistical significance were used. MATLAB scripts including these functions and other from the toolboxes already mentioned were developed for our group to accomplish the procedures outlined above. We also used STATISTICA software (http://www.statsoft.com/Products/STATISTICA-Features) for graphical and statistical purposes.

## 3. Results

### 3.1. Acoustic Features of Sound Stimulus

**Figure 1:**
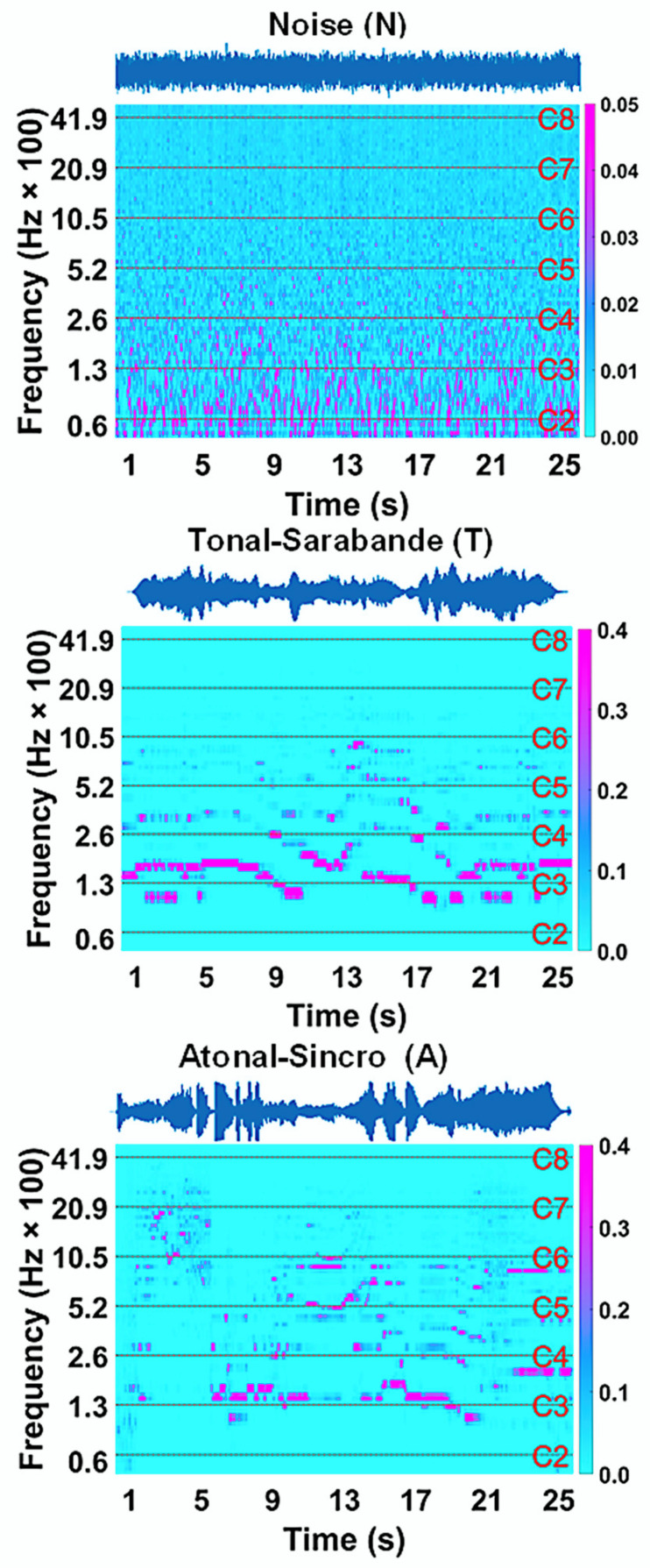
From top to bottom are: the acoustic signal/stimulus and time-frequency spectrum of the pink noise (N), baroque tonal Sarabande excerpt (T) and, contemporary atonal music excerpt Sincro (A). The dashed line in the spectra delimits the successive octaves of the piano keyboard; for each octave, the frequency level of the musical note C is indicated on the right of the frequency axis. Time axes in seconds (s) for signals and spectra, and color bar (right side) in relative power units.

Figure 1 shows the three acoustic signals (sound stimulus) corresponding to the pink noise (N), baroque tonal Sarabande (T), and contemporary atonal excerpts (A). Below each signal are plotted the time-frequency power spectra through which the temporal evolution of the spectral power distribution over the 26 s of stimulus emission can be followed. The time-frequency behavior for each stimulus is clearly different. In the case of N, it does not change over time, which is obvious from its construction, while the time-frequency behavior of T and A evolves differently because the power distribution per octave (from C2 to C8) is different.

**Figure 2:**
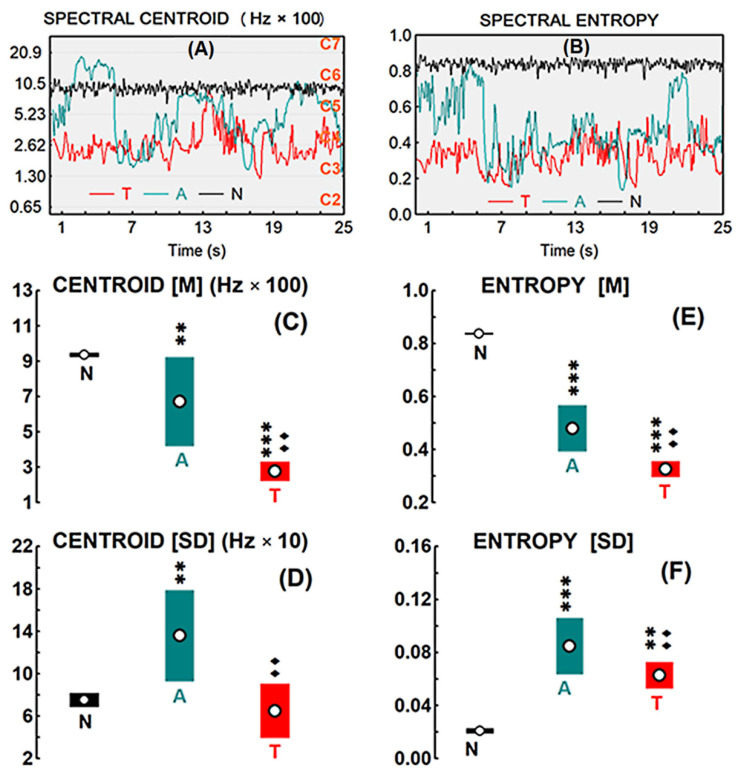
(**A**) Time evolution of the spectral centroid of each signal-stimulus [N for noise, A for atonal, T for tonal]; the frequency of the different octaves (C2 to C7 on the right) is indicated on the left. (**B**) Time evolution of spectral entropy. (**C**) Spectral centroid mean [M] and (**D**) Spectral centroid variability [SD] throughout the stimulus time. (**E**) Spectral entropy mean [M] and (**F**) Spectral entropy variability [SD] throughout the stimulus time. Open circles indicate the means, and color ranges the 95% confidence interval. Statistical significance levels of the differences between T or A against N were indicated by asterisks: (**) when *p* < 0.01, and (***) when *p* < 0.001. The same levels, but in solid diamonds (♦), were used for T against A.

As regards to the signals acoustic parameters analyzed, Figure 2A,B shows the spectral centroid (SC) and spectral entropy (SE) during the hearing period of each stimulus, respectively. Figure 2C gives the average of SC means (M) corresponding to the 13 two-second segments into which the hearing period was divided, and Figure 2D, the SC average of segments variability (SD). Figure 2E,F show the M and SD for SE, respectively. The K-W test was applied for the M of SC, since the variances between the three auditions were non-homogeneous (***). The test showed statistically significant differences [H (2, 39) = 24.89 (***)] between the three auditions. The post-hoc MWU test gave significant differences between N-A (**), N-T (***), and A-T (••). As for the variability SD of SC, the K-W test showed significant differences (***) between the three auditions [H (2, 39) = 14.03], and the post-hoc MWU showed significant differences between N-A (**) and A-T (••), but not between T-N. The K-W test was also applied for the results of the M of SE, as the variances in the three auditions were non-homogeneous (***). This test showed statistically significant differences [H (2, 39) = 29.97 (***)] between the three auditions, and the post-hoc MWU test showed significant differences between N-A (***), N-T (***), and A-T (••). As for the SD of SE, the K-W test was also applied and showed significant differences (***) between the three auditions [H (2, 39) = 26.60], and the post-hoc MWU showed significant differences between N-A (***) and N-T (**), but not between N-T.

### 3.2. Results of the Network Based Statistic (NBS)

**Figure 3:**
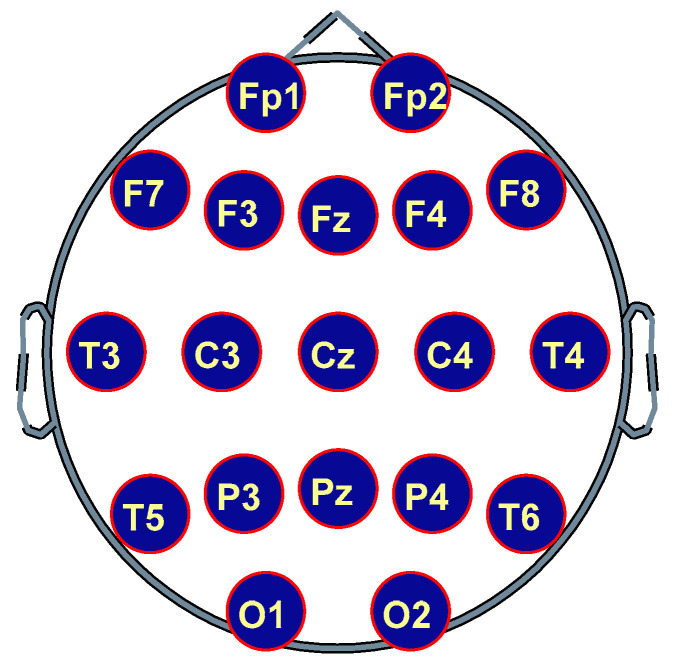
Superior plane of the EEG electrodes/graph nodes location.

Cortical areas underlying EEG electrode locations were used for topographic cortical designations of the EEG graph nodes; thus, prefrontal areas for Fp1/Fp2; prefrontal-ventrolateral for F7/F8, prefrontal-dorsolateral for F3/F4; interhemispheric frontal cortex for Fz and Cz; temporary cortical zones for T3/T4/T7/T8; parietals for C3/C4/P3/P4; parietal-interhemispheric for Pz and occipital cortical areas for O1/O2. Figure 3 shows the approximately location of the electrodes or graph nodes used in the results and discussion sections drawn from a superior plane view. In the NBS calculations, the connectivity matrices (non thresholded) that make up the graph or connectome EEG of each subject in each condition and BF were used.

#### 3.2.1. Between-Group Comparisons in Each Audition Separately

**Figure 4:**
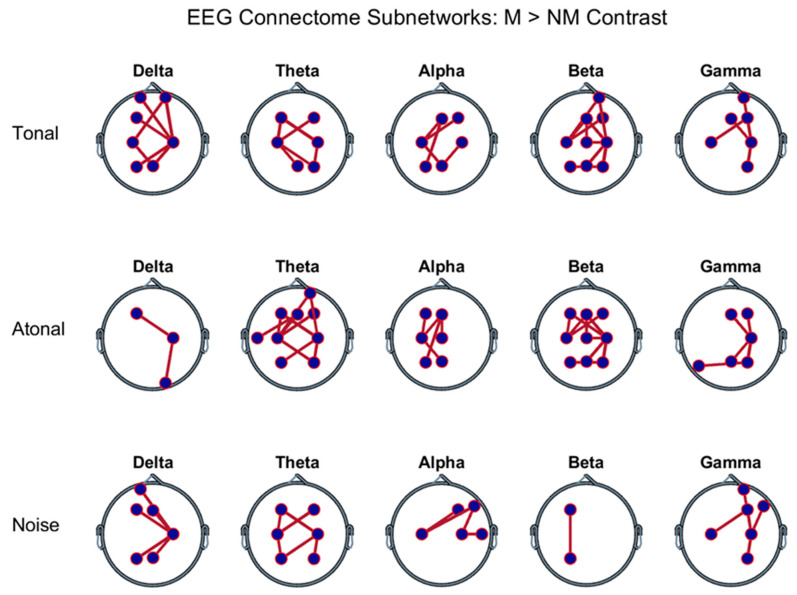
Electroencephalographic (EEG) connectome’s subnets that were significant (FDR < 0.05) when comparing musicians (M) versus non-musicians (NM). They are plotted in the frequency bands (in columns) and during the auditions indicated in each row on the left. The connections (red lines) between nodes (blue circles) that make up each subnetwork correspond to functional connectivities whose magnitude was higher in the M than in the NM. See subnetworks statistical significance in the text.

The results shown in Figure 4 reflect the existence of a subnet of the EEG-CF graph/network formed by a certain number of connections in which the FC was higher in musicians (M) than in non-musicians (NM); this occurred in each of the music auditions [tonal (T), atonal (A), and noise (N)] and for each of the EEG frequency bands analyzed. In each FB, the levels of statistical significance during the music auditions T, A, and N were respectively: *p* = 0.001 ± 0.001, *p* = 0.005 ± 0.003, and *p* = 0.001 ± 0.001 in the delta band; *p* = 0.001 ± 0.001, *p* = 0.001 ± 0.001, and *p* = 0.000 ± 0.000 in the theta band; *p* = 0.000 ± 0.000, *p* = 0.001 ± 0.001, and *p* = 0.001 ± 0.001 in the alpha band; *p* = 0.000 ± 0.000, *p* = 0.000 ± 0.000, and *p* = 0.009 ± 0.004 in the beta band; and *p* = 0.001 ± 0.001, *p* = 0.002 ± 0.002, *p* = 0.001 ± 0.001 in the gamma band. The calculation parameters used in the NBS to obtain the above results were: FDR level less than 0.05, number of permutations equal to 2000, and intensity as a component (sum of T values). In all FBs, there was no exact match between the 3 auditions, neither in the number of connections nor between the interconnected nodes. In delta FB, the node with greatest degree in the 3 auditions was C4, but the number of connections was greater in T than in A and N and greater in N than in A. In FB theta, the node with greatest degree in the 3 auditions was C3, but the number of connections was greater in A than in T and N and similar in T than in N, but without connections inter-nodes identical. In FB alpha, the node with the greatest degree in the 3 auditions was C3 and the number of connections (6) was equal in T, A, and N although the connections inter-nodes was not identical in the three cases. In beta FB, the greatest degree nodes were C3 and C4 in T and A; but in N, the number of connections was lower in N than in T and A; besides, in the 3 auditions, the inter-node connections were different. In FB gamma, the node with the greatest degree in the 3 auditions was C4 and, as in alpha, the number of connections (6) was similar in T, A, and N although the inter-node connections were different in the three cases. The nodes that least intervened in all FBs and auditions were the prefrontals-ventral, the temporals, and the occipitals.

Connections pointed out in Figure 4 were: (a) in tonal-music, delta-band: C3-Fp2, Fp1-C4, F3-C4, P3-C4, Fp2-C4, C3-Pz, C4-Pz; theta-band: F3-C3, C3-F4, F3-C4, C3-P4, C4-P4, C3-Pz; alpha-band: C3-F4, C3-Fz, P3-Fz, C3-Pz, C4-Pz; beta-band: C3-Fp2, C3-F4, Fp2-C4, P3-P4, C4-P4, C3-Fz, C4-Fz, C4-Cz, Fz-Cz, C4-Pz; gamma-band: C3-F4, Fp2-C4, F4-C4, C4-P4, C4-Fz; (b) atonal-music, delta-band: F3-C4, C4-O2; theta-band: F3-C3, C3-Fp2, C3-F4, F3-C4, P3-C4, Fp2-C4, C3-P4, C4-P4, C3-Fz, T3-Fz; alpha-band: F3-C3, C3-Fz, P3-Fz, Fz-Cz, C3-Pz; beta-band: F3-C3, C3-F4, F3-C4, P3-P4, C4-P4, C3-Fz, C4-Fz, C4-Cz, Fz-Cz, C4-Pz; gamma-band: F4-C4, T5-P4, C4-P4, C4-Fz, C4-Pz; (c) noise sound, delta-band: Fp1-C4, F3-C4, P3-C4, C4-Fz, C4-Pz; theta-band: F3-C3, C3-P3, C3-F4, F3-C4, P3-C4, C4-P4; alpha-band: C3-F4, C3-F8, F8-C4, C4-T4; beta-band: F3-P3; gamma-band: C3-F4, Fp2-C4, F4-C4, F8-C4, C4-P4.

#### 3.2.2. Pairwise Comparisons between Auditions in Each FB and in Each Group Separately

In none of the FBs considered and for neither of the two groups of subjects did we find significant differences in the paired contrasts between the auditions performed.

### 3.3. Results from Graph Metric

#### 3.3.1. Global Centrality Measures of EEG Graph

**Figure 5:**
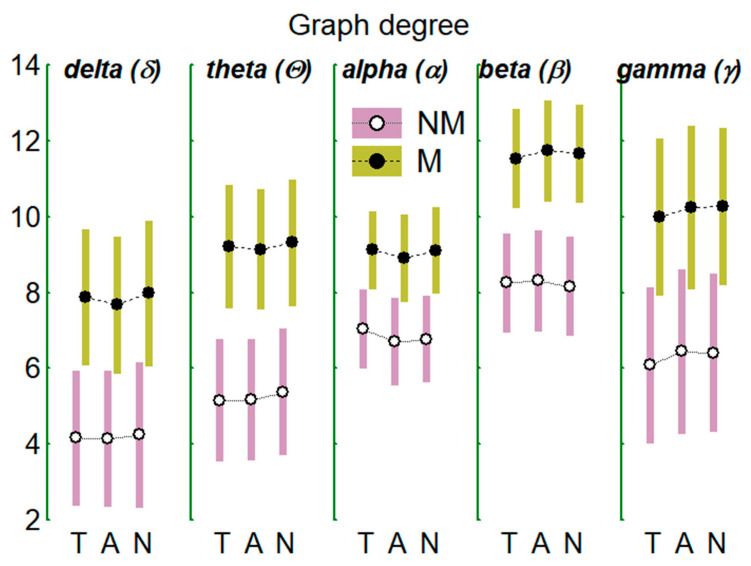
Mean values (±95% confidence interval) for the degree of EEG graph/network in the two groups of subjects (NM and M) in the 5 indicated EEG frequency bands and in the 3 auditions considered (T: tonal, A: atonal, and N: noise).

**Figure 6:**
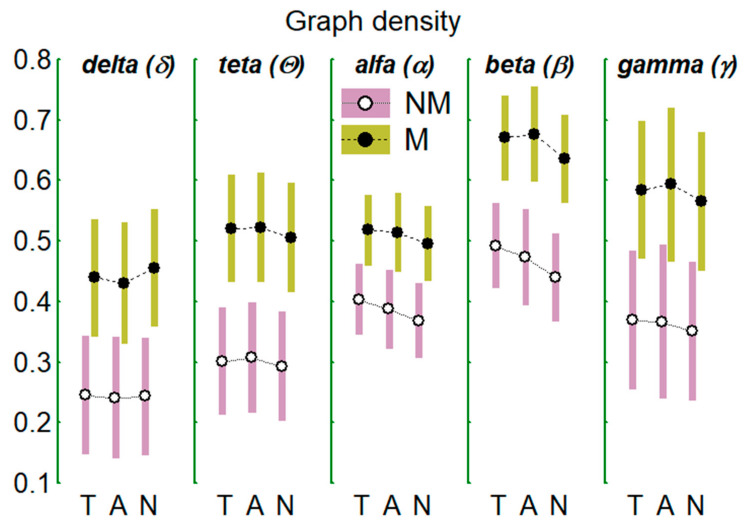
As in Figure 5 but for the density of the EEG graph.

Table 1 shows the results of the MANOVA test corresponding to the centrality indices of the graph: degree, density, and strength. The table shows the statistical results (F statistic, DF degrees of freedom, and significance level P) for the global comparison between groups (MNM) and of the multivariate (W-R) or univariate (L-B) tests for the repeated factors (within-groups), that is, the 5 frequency bands (FB), and the 3 auditions (TAN), T, A, and R. In addition, interactions between the factors results (e.g., FB * TAN) are shown when they were significant. For the sake of clarity, only those factors or interactions that were statistically significant are shown. The last column (Comp.) discloses the results of between groups (NM vs. M) comparisons and of the post-hoc tests between repeated factors and interactions. Regarding graph degree, between-groups differences appear: musicians clearly show a greater nodal degree than non-musicians; this effect also appears for the other two indices (intensity and nodal density). Degree and density (but not the strength) change with the FB considered being of greater magnitude in the beta (β) band as expressed in the Table 1 and Figure 5 and Figure 6. Changes between auditions were only recorded for the graph density index. Independently of the groups (or what is the same considering both groups as one) and independently of the frequency bands (all bands), the density in T was greater than in N; it was also greater in A than in N; however, there were no differences between T and A. In addition, the differences between auditions when the FBs are considered separately and both groups as only one (FB * TAN interaction), only appear in the band beta being the graph density in this band greater in T than in N (Figure 6). The alterations of the indices mentioned with FB and TAN, when occur, always do so in both groups, that is, they occur independently of the group type.

#### 3.3.2. Efficiency Indices of the Internode Communication of the EEG Graph

**Figure 7:**
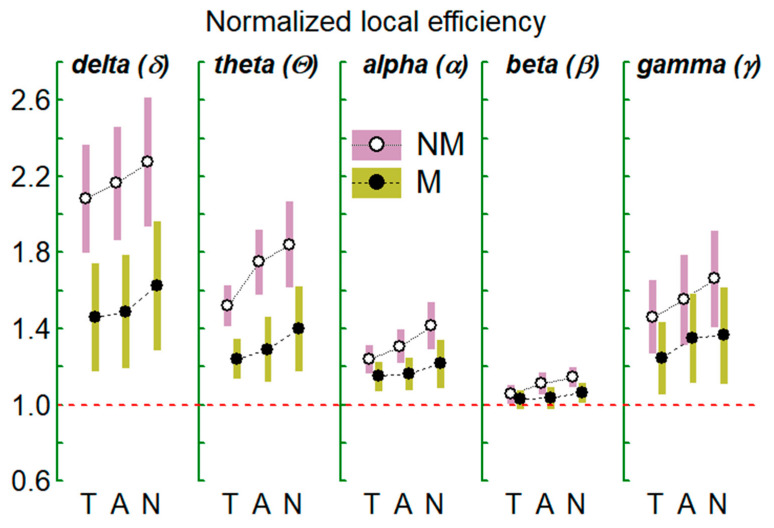
As in Figure 5 but for the normalized local efficiency of the EEG graph/network. The red line indicates the limit value for a random network according to the selected normalization process.

**Figure 8:**
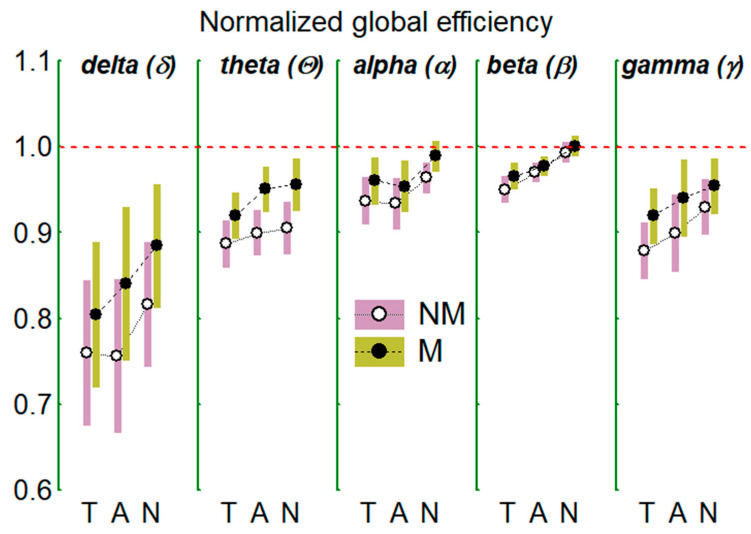
As in Figure 7 but for the normalized local efficiency of the EEG graph/network.

In Table 2, the MANOVA results are shown for the normalized (vs. random network) local efficiency (NLE) and for the normalized (vs. random network) global efficiency (NGE) and in Figure 5 and Figure 6 are shown the indices mean values for the two groups (NM and M), three conditions (T, A, and N) and five FBs. Table 2 structure and captions are as Table 1. Here, the magnitudes of the normalized global efficiency (NGE) was (regardless of FB and of audition considered) statistically greater for the Ms than for the NMs, while the normalized local efficiency (NLE) resulted greater for the NMs than for the Ms. Furthermore, regardless of the groups and auditions, NGE reached its maximum value in the EEG beta-network as did the graph degree and density; on the contrary, in this beta network, NLE reached its minimum value (see also Figure 7 and Figure 8). Alterations with auditions (regardless of MNM and FB) were found for both indices: their values were different among the three auditions being in T < A, in T < N, and in A < N. Moreover, NLE and NGE magnitudes were closest to those expected for a small-world (SW) type network mainly in the EEG beta-network (NGE ~ 1 and NLE > 1, Watts and Strogatz, 1998); additionally, it can be observed (see Figure 7 and Figure 8) that, going from T to N, NLE moved away from 1 (i.e., increases its local information transfer relative to that of a random network) while NGE (the global information transfer indices) increase and approaches to 1 (i.e., to a random network). On the other hand, this SW structure appears to be more prominent for Ms group than the NMs. Finally, NGE exhibit FB*TAN interactions: clear differences in the delta band network between T-A and T-N and, in the beta and gamma networks between T-N.

#### 3.3.3. Results of the Graph Metric at the Node Level Topographic Maps

**Figure 9:**
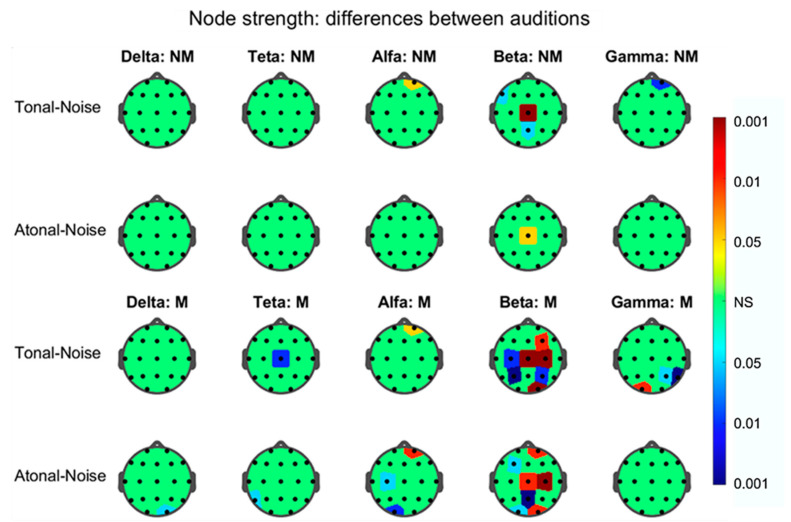
Statistical significance values (P) of the difference of the node degree between tonal-noise and atonal-noise auditions (left column) for the different nodes of the five graphs of each EEG frequency band pointed out. The color of the area around each node corresponds to the significance level P indicated in the color-bar on the right (valid to all heads); the colors were associated with the three levels of significance (*p* < 0.001, *p* < 0.01, and *p* < 0.05) by using three range of reds when the difference was positive and three range of blues when it was negative. Nodes without statistical significance (NS) appear in green. In the upper two rows the values for non-musicians (NM) appear in the indicated frequency bands and in the lower two those corresponding for musicians (M) group.

**Figure 10:**
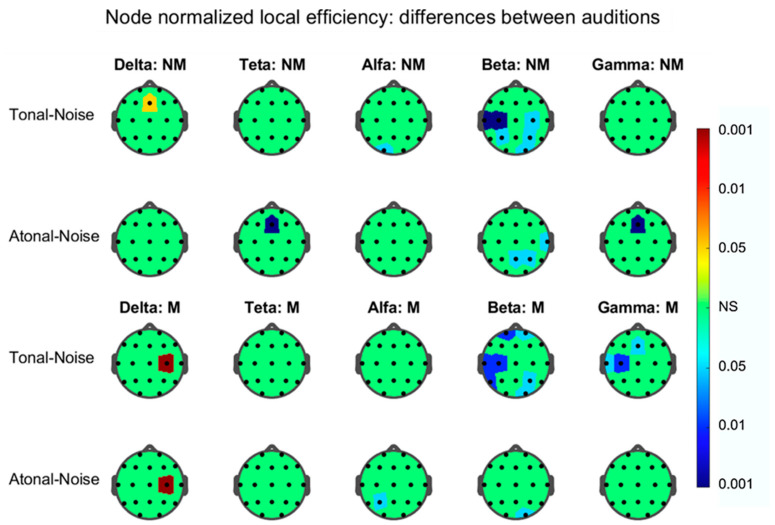
As in Figure 9 but for the normalized local efficiency.

The results for graph degree, strength, and normalized local efficiency—calculated for the nodes of each FB-EEG graph in each audition—showed statistically significant differences between tonal-noise and atonal-noise auditions only, for the strength and efficiency indices in both groups of subjects and mainly in the graph corresponding to the beta band. These results are depicted in Figure 9 and Figure 10.

As for the node strength index (Figure 9), the nodes <> exhibiting significant differences were the following: (a) for the NMs in the tonal-noise contrast: in the alpha graph, <F2>, in the beta graph, <F6>, <Cz>, and <Pz>; in the gamma graph <F2>; in the atonal-noise contrast: in the beta graph <Cz>; (b) for the Ms, were in the tonal-noise contrast, in the theta graph, <Cz>, in the alpha graph, <F2>; in the beta graph, <F4>, <C3>, <Cz>, <C4>, <P3>, <P4>, and <O2>, in the gamma graph <P4>, <T6>, <O1>; in the atonal-noise difference, in the delta graph, <O2>, in the theta graph, <T5>, in the alpha graph: <C3>, <O1>, <F2>, and in the beta graph, <Fp2>, <F3>, <Cz>, <C4>, <Pz>, <O1>, and <O2>. Therefore, from the previous results, the beta-band EEG graph was the plot in which more clearly the differences among auditions were observed that occurred in both subjects groups. Thus, in this graph for the NMs, only a few nodes appear significant in the T-N contrast and even lesser (Cz) en the A-N; however, for the Ms, seven nodes were implicated in T-N and also seven in A-N but it did not exist complete coincidence between them.

In relation to the normalized local efficiency (Figure 10), the results were similar to those found with the nodal strength. Thus, the graph in which the differences between auditions appeared most prominent was the one corresponding to the beta band. In this beta graph and in the T-N contrast of the NMs, the nodes T3, C3, C4, P3, P4, and O2 were significant and, in the A-N contrast, the nodes T4, Pz, and P4; in the Ms in the T-N contrast, the significant nodes were Fp1, Fp2, T3, C3, T5, P4, and O2 and in A-N, only node O2. Therefore, for this graph and for both groups of subjects, the number of nodes involved in the T-N contrast was greater than that of the A-N contrast. In both groups, and in the T-N contrast, there was no coincidence in the significant nodes: Thus, e.g., in the Ms (T-N contrast) the nodes Fp1 and Fp2 appear involved, which did not appear in the T-N contrast of the NMs.

#### 3.3.4. Results from Between-Group Differences Comparisons during Auditions

**Figure 11:**
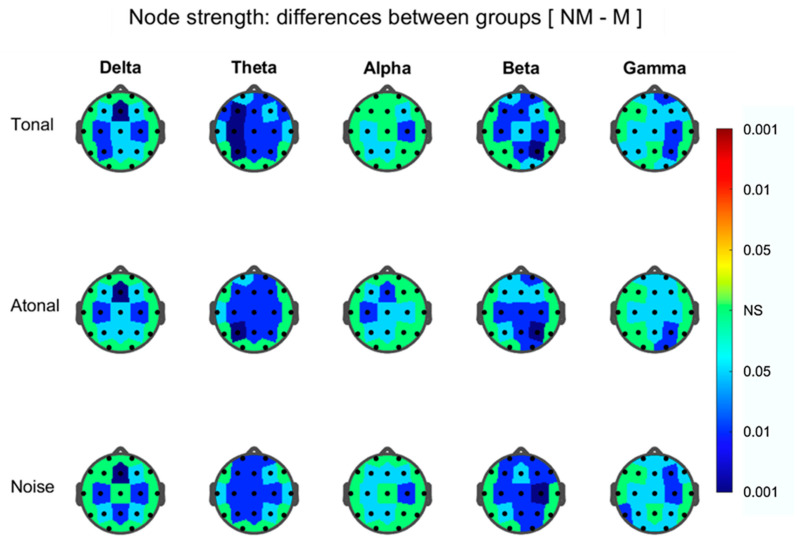
As in Figure 9 but for the difference between non-musicians (NM) and musicians (M) of node strength, during the three auditions indicated at left.

**Figure 12:**
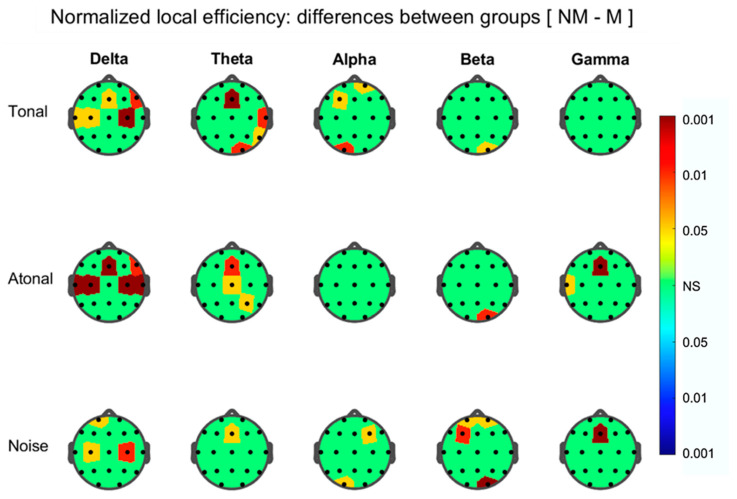
As in Figure 9 but for the difference between non-musicians (NM) and musicians (M) of node normalized local efficiency, during the three auditions indicated at left.

Node degree results show the same trend than those found in the NBS during the three auditions. Here, for all FB’s graphs, the majority of the nodes (at T, A, and N) presented a connectivity that was greater for the Ms than for NMs. This occurs essentially for prefrontal, prefrontal (dorsal), frontals, and parietals areas (Figure 11) where no clear differences among auditions are present. Between-group results for the NLE (Figure 12) show that the nodes which exhibit significant group differences (in all FB graphs), were much lower than for the nodal strength and with the NLE greater in the NMs than for Ms. Thus, in the beta graph node occipital O2 reflect group, differences (NM > M) during T, A, and N but during N, also were additionally implicated (NM > M) the prefrontal nodes Fp1, Fp2, and F3.

## 4. Discussion

### 4.1. Common Features to Musicians and Non-Musicians during Auditions

The results for the global central indices of the graphs that model the networks formed by the EEG functional connectivities (FC) between electrodes/nodes in the different frequency bands (FB) (δ, θ, α, β, γ-network) show that the network in which the degree and density appear more salient was the β-network, while the network that exhibits the lowest values of these indices was that of the δ-network (see Table 1 and Figure 5 and Figure 6). Another feature common to all networks, was that the magnitude of graph density during the audition of tonal music (T) and of atonal music (A) was greater than that obtained during noise (N) although indistinct between T and A.

In relation to the normalized efficiency indices that assess the local (NLE) and global (NGE) transmission of information (see Table 2 and Figure 7 and Figure 8), the β-network was the network in which both indices more closely approximate the expected values for a small-world network (SW) (NLE> 1 and NGE ~ 1) and, the δ-network, the one in which they were farthest from SW. Moreover, these indices, independently of the FB and the groups, showed different magnitudes in the three auditions: In the case of local efficiency, it was greater but close to 1 during T but increased significantly during A and even more during N. On the other hand, the global efficiency was close to 1 during N but significantly lower during A and lower during T than in A. Specifically, in the β-network and during the three auditions, the efficiency values are close to that of a SW structure but with a different degree of approximation according to the audition considered. In effect, the efficiency in transmission of information between nearby nodes/clusters of the network (NLE) is low and close to that of a random network (RN) during T, but increases during A and N. On the other hand, the efficiency in the global transmission between distant centers (NGE) is close to that of an RN (in which is considered optimal) during N but decreases during A and is even smaller in magnitude in T. Therefore, it seems that during the tonal music audition of less timbre complexity (entropy), as shown by the results of the acoustic analysis, and musically with a harmonic structure and ternary measure and socially and culturally more familiar, requires less cognitive effort (lower NLE and NGE) than atonal music, which with a more complex timbre, musically unstructured and with less spread and social acceptance, demands a higher cognitive effort (greater NLE and NGE). Our results are in agreement with those reported (for EEG connectivity network in alpha band) during Chinese music listening [40,75], talking about that music listening produces an enhancement of SW network organizations. This work also contributes, that different styles of music of different timbre brightness and complexity (and musical structure and syntax) produce appreciable different alteration in the brain organization SW, during the noise audition when the organization of the network most resembles that of a RN. Moreover, the fact that EEG-FC networks indices are different according to the FB seems to be expected if we consider that different EEG-FBs are associated with different sensory and cognitive components [7,76] and with the activity of musical perception of different brain cortical and subcortical structures [22,77,78]. On the other hand, we must also highlight that in the networks corresponding to lower frequency bands (especially delta), the values of NLE and NGL are far from that of the SW network organization, although their magnitudes remain in the order T < A < N. In effect, in the delta-network during N audition, NLE reaches it maximum and NGE its minimum. Although, we find no clear explanation for this strange behavior, we will discuss some factors that can help us understand it. From the perspective of acoustic characteristics of the stimuli, the noise with no defined timbre, with its centroid (SC) located at the average frequency of the interval considered, with a very high entropy (SE), and with low SC or SE variability (Figure 2) constitutes a sound without structure (without harmony or rhythm) and without changes during the audition. Therefore, at most, its audition can only produce a state of alert or continuous strangeness and even unconsciousness. The fact δ-network exhibits greater local efficiency during unstructured sounds (N) than during A (music without tonal/rhythm structure but with academic style and underlying structure) and still higher than during T music, may be due to the complex structure of this delta network, possibly caused by its subcortical connections, which are also at the origin of these slow EEG waves and that it also affects brain connectivities in this band. Indeed, delta waves appear during the deep sleep of mammals when there seems to be an effective disconnection of the cortex due to changes in the activity of cortical neurons) [79,80]. In this line, the EEG of certain animals lacking a true neocortex, such as reptiles, is known to exhibit slow activity in the delta range [81]. On the other hand, and in general, it is known that long-distance networks that connect cortical and subcortical zones operate in slow FB (delta to alpha range), while cortical networks manifest themselves in faster FB, such as beta or gamma [82]. Therefore, delta wave cortical activity and FC networks in humans seems to be more related to the dynamics of subcortical neuronal structures of greater evolutionary age, with substantial local efficiency or communications between neighboring neural clusters and lower efficiency in the information transfer between distant neural centers as happen during sleep. Furthermore, this delta-network organization (including its lower node degree and density) makes it more evolutionarily prepared to respond to unstructured sounds such as noise or A than to structured music such as T.

### 4.2. Differences between Auditions in Each Group Separately

The strength and local efficiency NLE topographic maps at the nodal level show that these graph indices present nodes in which the T-N difference is not coincident with the A-N. This effect is mainly observed in the beta band graph and, furthermore, the nodes involved in each difference do not match in both groups as can be seen in Figure 9 and Figure 10. Thus, in the strength beta graph for the NMs, only a few nodes (*n* = 3) appear significant in the T-N contrast and even lesser (*n* = 1) in the A-N contrast; however for the Ms, seven nodes were implicated in T-N and also seven in A-N but without it existing a complete match between them. As for the NLE beta graph and for both groups of subjects, the number of nodes involved in the T-N contrast was greater than that of the A-N contrast. In both groups and in the T-N contrast, there was no coincidence in the significant nodes; thus, e.g., in the Ms (T-N contrast), the prefrontal nodes Fp1 and Fp2 appear involved, which did not appear in the T-N contrast of the NMs. Therefore, these results confirm and expand those related in the previous section showing the beta band graph, as the network structure in which the results of the graph indices are best visualized and in which differences between the T-N and A-N contrasts appear more clear in both groups of participants.

### 4.3. Between-Group Differences during Auditions

The different graph indices showed clear between-groups differences (M vs. NM) in all FB networks analyzed corresponding to the three auditions. In addition, in some networks, M-NM difference was also different according to the auditions. Regarding the NBS results, in all the graphs, there were significant subnets with dissimilar number of connections and in which the FC was greater in the M than in the NM (Figure 4). For the five FB subnets and the three ones corresponding to each audition, no coincidence neither with respect to the number of connections or with respect to interconnected regions was found. The most common connections in the different FBs and auditions subnets were between bilateral dorsal prefrontal regions, interhemispheric-central frontals, and bilateral parietal regions (Figure 4). There was no pattern that allowed us to associate or distinguish the number and the interconnected brain areas in the 5 FB subnets or between the subnets of the 3 auditions for each FB.

Global indices of the graphs, degree, density, strength, and NGE efficiency, were for all FB and the three auditions greater in the Ms than in the NMs except for the NLE index that was greater in the NMs than in the Ms. These indexes did not show in any case significant interactions between the groups and the FBs or between the groups and the auditions. However, the results corresponding to the topographic maps of the nodal strength (Figure 11), do show for certain nodes of each FB graph, between-group differences (always with M > NM) without there being a total match in the affected nodes of each FB graph. Thus, for example, when we consider the results for T music in the frontal lobe in the alpha band, only the right prefrontal-dorsal node appears significant (M > NM) while in the beta band, the nodes bilateral prefrontals, prefrontals-dorsals, and interhemispheric frontals appear with M > NM. On the other hand, when we consider the results corresponding to the frontal lobe in the three auditions (T, A, and N), e.g., in the alpha band, we find that in T, only the right prefrontal-dorsal zone appears with M > NM, but in A, the left prefrontal-dorsal zone and the interhemispheric frontals appear with M > NM, and in N, were the prefrontals-dorsals bilateral and an interhemispheric frontal. In other words, the topographic maps of nodal strength show us cortical zones in which the node strength is greater in the Ms than in the NMs, but which differ according to the FB and according to the audition considered.

Regarding the local efficiency NLE, only a few nodes are involved in each graph in which the index is greater in the NMs than in the Ms and that are different in the different FB networks and for the same FB they appear differ in the three auditions (Figure 12). In conclusion, the Ms (in relation to the NMs) exhibited, in the EEG FC networks of the different FBs, and, in the three auditions considered (a) subnets with greater FC than the NM; (b) greater global degree, density, and strength than the NMs, and (c) a greater global efficiency in relation to that of the NMs, therefore a better efficiency in the transfer of information between distant brain regions, but instead, they show less efficiency in neural communication between nearby zones than that of NMs.

We did not find in the literature works differences between Ms vs. NMs in the context of the present work. However, in neuroimaging (fMRI) and neuropsychology studies, in general, it has been reported that the differences between both groups may have to do with the greater efficiency of the attention network shown by the M vs. the NM: the Ms seem to have a greater efficiency with respect to the executive attention network [83]. Along these lines, certain areas of the bilateral dorsomedial and dorsolateral cortex have been related to cognitive functions and abilities of memory and attention [84,85], these effects would justify the greater extension of the FC subnets in M vs. NM during T and A than during N in certain FB networks, which we found in the NBS results. There are research works in which it is demonstrated that musical experience seems to influence the functional connectivity (EEG) of some cortical areas, such is expressed in our NBS and topographic map results. Indeed, in expert musicians, listening to extracts of tonal music modified the magnitudes of spectral coherence of the EEG in the alpha and beta bands with respect to non-musicians [39] and the phase synchronization of the gamma band, especially the left hemisphere [25]. Furthermore, the phase synchronization of expert musicians was greater than that of non-musicians listening to the same musical extract [23]. Moreover, in agreement with our results, Ms in regard to NMs, exhibit stronger FC than NMs between the primary auditory cortex, the primary motor cortex, and in the right ventral premotor cortex [86], also a significantly higher density of local functional connectivity in different brain regions [87], and a greater insular and parietal opercular connectivity [88]. Finally and partially confirming our results it has been reported that functional coupling between motor and auditory areas appears to be modulated as a function of musical training [89].

## 5. Conclusions

Two measurements—the normalized local and global efficiencies—used to explore how efficiently the network exchanges information, were able to discriminate both styles irrespective of groups and frequency bands. However, it was the beta-band network that reflected that during the tonal audition, its structure was close to that of a network with a small-world organization, while the atonal music and noise produced a moving away from this organization. This effect was attributed to the different timbre characteristics of the sounds listening and to their different harmonic and rhythmic structure. The topographic maps (especially that of the beta band) of the graph-networks corresponding to the nodal force and the local efficiency also allowed us to find that the difference between tonal music and noise, and between atonal music and noise, for the set of graph nodes, did not coincide, that is, they thus involved different cortical zones. For the same FB and for the same contrast between sounds, those differences did not coincide between musicians and non-musicians. Differences between musicians and non-musicians using the NBS procedures were also found: subnets formed only by certain connections between different cortical zones showed a FC greater in the musicians but with different structure in all FB and for the different auditions.

## Figures and Tables

**Table 1:** MANOVA results for the EEG graph indices, degree, density, and strength. In the first column, the factors indicated were: (MNM for comparisons between musician (M) vs non-musicians (NM) groups, FB for repeated comparisons between frequency bands, TAN for those corresponding to the different auditions [tonal (T), atonal (A) and noise (N)], and FB * TAN for cross-interactions; in successive columns, appear the test used (W-R or L-B); the F statistic, the degrees of freedom (DF), and the level of significance P; in the last column (Comp.) with asterisks * the significance levels of the a posteriori comparisons between pairs of repeated factors are indicated (** for *p* < 0.01, and *** for *p* < 0.001).

Factors	Test	F	DF	*p*	Comp.
**Graph degree**					
MNM		12.16	1.30	0.001	M > NM
FB	L-B	24.56	1.30	0.000	β > δ ***; β > θ ***; β > α ***; β > γ ***
**Graph density**					
MNM		12.13	1.30	0.001	M > NM
FB	L-B	26.42	1.30	0.000	β > δ ***; β > θ ***; β > α ***; β > γ ***
TAN	W-R	12.59	2.29	0.000	B > R ***, C > R **
FB*TAN	L-B	4.51	1.30	0.042	(β): B > R ***
**Graph strength**					
MNM		11.67	1.30	0.001	M > NM

**Table 2:** Captions and table structure are as those of Table 1 but for the MANOVA of the normalized local and global efficiency indices. (* for *p* < 0.05, ** for *p* < 0.01, and *** for *p* < 0.001).

Factors	Test	F	DF	*p*	Comp.
**Normalized local efficiency**					
MNM		13.38	1.30	0.000	M < NM
FB	L-B	31.03	1.30	0.000	β < δ ***; β < θ ***; β < γ ***
TAN	L-B	22.79	1.30	0.000	T < A **; T < N ***; A < N **
**Normalized global efficiency**					
MNM		4.82	1.30	0.036	M > NM
FB	L-B	31.45	1.30	0.000	β > δ ***; β > θ *; β > γ *
TAN	W-R	41.94	2.29	0.000	T < A ***; T < N ***; A < N ***
FB*TAN	L-B	4.87	1.30	0.035	(δ) T < A ***; T < N ***; (β) T < N *; (γ) T < N *

## Data Availability

The data presented in this study are available on request from the corresponding author.

## Supplementary Materials

The following are available online at https://www.mdpi.com/2076-3425/11/2/159/s1. The musical excerpts included in the work can be heard in the way indicated by the editor. We enclose the files of the extracts “sarabande.wav”, “sincro.wav” and “noise.wav” as supplementary material.
